# Lung toxicity and biodistribution of Cd/Se-ZnS quantum dots with different surface functional groups after pulmonary exposure in rats

**DOI:** 10.1186/1743-8977-10-5

**Published:** 2013-03-04

**Authors:** Jenny R Roberts, James M Antonini, Dale W Porter, Rebecca S Chapman, James F Scabilloni, Shih-Houng Young, Diane Schwegler-Berry, Vincent Castranova, Robert R Mercer

**Affiliations:** 1 Pathology and Physiology Research Branch, Health Effects Laboratory Division, National Institute for Occupational Safety and Health, Morgantown, WV 26505, USA

**Keywords:** Nanoparticles, Cadmium, Toxicology, Lung, Tissue distribution

## Abstract

**Background:**

The potential use of quantum dots (QD) in biomedical applications, as well as in other systems that take advantage of their unique physiochemical properties, has led to concern regarding their toxicity, potential systemic distribution, and biopersistence. In addition, little is known about workplace exposure to QD in research, manufacturing, or medical settings. The goal of the present study was to assess pulmonary toxicity, clearance, and biodistribution of QD with different functional groups in rats after pulmonary exposure.

**Methods:**

QD were composed of a cadmium-selenide (CdSe) core (~5nm) with a zinc sulfide (ZnS) shell functionalized with carboxyl (QD-COOH) or amine (QD-NH_2_) terminal groups. Male Sprague–Dawley rats were intratracheally-instilled (IT) with saline, QD-COOH, or QD-NH_2_ (12.5, 5.0, or 1.25 μg/rat). On days 0, 1, 3, 5, 7, 14, and 28 post-IT, the left lung, lung-associated lymph nodes (LALN), heart, kidneys, spleen, liver, brain, and blood were collected for metal analysis of Cd content by neutron activation to evaluate clearance and biodistribution. One right lobe was ligated and fixed for microscopy and histopathological analysis. The remaining right lobes from rats in each group were subjected to bronchoalveolar lavage (BAL) to retrieve BAL fluid and cells for analysis of injury and inflammation.

**Results:**

Lung injury and inflammation was found to be dose-dependent and peaked at days 7 and 14 post-exposure for both forms of QD, with slight variations in degree of toxicity at early and later time points. Both QD appeared to lose their fluorescent properties and destabilize after 1 week in the lung. Cd persisted up to 28 days for both forms of QD; however, clearance rate was slightly greater for QD-COOH over time. No Cd was detected in the liver, spleen, heart, brain, or blood at any time point. Cd appeared in the LALN and kidneys beginning at 1–2 weeks post-exposure.

**Conclusions:**

QD-COOH and QD-NH_2_ differed in clearance rate and differed slightly in degree of toxicity at different time points; however, the overall pattern of toxicity and biodistribution was similar between the two particles. Toxicity may be dependent on the dissolution rate and bioavailability of free Cd.

## Introduction/Background

Quantum dots (QD), also referred to as nanocrystals, are fluorescent semiconductor nanomaterials (~2-100 nm in diameter) commonly used in the biomedical and electronic industries [1,2]. In terms of structure, QD are nanocrystals composed of a semiconductor core [e.g., cadmium-selenide (CdSe)] encased within a shell comprised of a second semiconductor material [e.g., zinc sulfide (ZnS)] [3]. Due to their small size, QD have unique optical and electronic properties that impart the nanoparticle with a bright, high intensity fluorescence [4], which persists as long the QD remain stable. The emission wavelength of the QD is specific to the size and composition of the nXanocrystal, where increasing size results in increasing emission wavelength. Because of their large surface area, QD are readily functionalized with targeting ligands for site-directed activity. Biomedical applications exploit the fluorescent properties of QD for diagnostic tools (e.g., imaging), therapeutic purposes (e.g., drug delivery and cancer treatment), live cell labeling, and tracking over long periods of time [2]. QD are also being incorporated into solar cell and electronic devices, such as LEDs, where their electronic and optical properties provide advantages.

Due to the presence of Cd, a highly reactive and potentially toxic metal, there is concern about the health risks associated with exposure to QD. *In vitro* studies indicate that some QD are cytotoxic in short-term, cell culture-based assays (as reviewed by [1,3]). QD composition, size, charge, exposure concentration, outer coating bioactivity (e.g., treatment with functional groups) and oxidative properties, and mechanical stability have been implicated as possible causative factors in the cytotoxicity of QD [1]. Potential conditions of exposure to QD include therapeutic, diagnostic, environmental, and occupational [1]. Workplace QD exposures (e.g., engineers, researchers, clinicians) may result from inhalation, ingestion, and dermal contact.

Concerning inhalation, numerous animal studies have examined the toxicology of other nanoparticles after pulmonary exposure [5]–[9]; however, little is known about the lung toxicity of QD. Ma-Hock et al. [10] performed a short-term (6 hr/day for 5 days) inhalation study in which Wistar rats were exposed to 4.1 mg/m^3^ (0.52 mg Cd/m^3^) of a liquid aerosol of Cd-based QD (cadmium sulfide core with a cadmium hydroxyl shell). A local inflammatory response was observed at 8 days after exposure that regressed after a 3-week recovery period with limited translocation of Cd from the lungs to systemic organs. In addition, QD were observed within phagolysosomes of alveolar macrophages (AM) after inhalation exposure. Clift et al. [11,12] demonstrated that QD, functionalized with an organic coating or COOH or NH_2_ (PEG) (polyethylene glycol) moieties, can cause oxidative stress, leading to modulated Ca^2+^ signaling in a macrophage cell line after prolonged *in vitro* exposure to sub-lethal concentrations, and that toxicity is largely dependent on surface coating.

The goal of the current study was to expand upon the limited information that is known about the *in vivo* effects after pulmonary exposure to QD. Pulmonary toxicity and lung distribution, clearance, and translocation of QD functionalized with different surface moieties, resulting in different surface charges, were examined. Commercially-available QD, composed of a CdSe (~5 nm diameter) core with a ZnS shell, were used. The QD were functionalized with either amine (QD-NH_2_) or carboxyl (QD-COOH) groups to form positively- or negatively-charged QD, respectively. Rats were intratracheally instilled with various doses of QD-NH_2_, QD-COOH, or phosphate-buffered saline (PBS; vehicle control). Lung injury and inflammation were measured at 2 hr and at 1, 3, 5, 7, 14, and 28 days post-exposure. Sites of particle deposition and distribution in the lungs and particle uptake by macrophages were assessed using autometallographic silver enhancement of QD in tissue, laser scanning confocal microscopy (LSCM), and transmission electron microscopy (TEM). Particle translocation from the lung was evaluated using neutron activation analysis of Cd in lungs, blood, heart, lung-associated lymph nodes (LALN), brain, kidneys, liver, and spleen.

## Results

**Figure 1:**
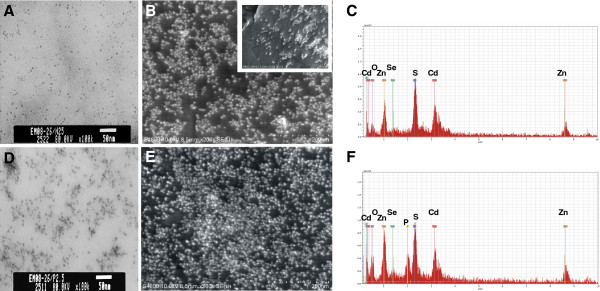
**QD characterization.** TEM micrographs of carboxyl-functionalize quantum dots (QD-COOH) (**A**) and amine-functionalized quantum dots (QD-NH_2_) (**D**). FESEM micrographs of QD-COOH (**B**) and QD-NH_2_ (**E**), including a high magnification FESEM image of QD-COOH (**B**: inset). The corresponding elemental mappings of QD-COOH (**C**) and QD-NH2 (**F**) were analyzed using energy dispersive X-ray (EDX) analysis at 20 keV (Cd, Cadmium; O, Oxygen; P, Phosphorous; S, Sulfur; Se, Selenium; Zn, Zinc).

Electron microscopy and EDX were performed to determine particle size and chemical composition of the QD samples (Figure 1). TEM and FESEM confirmed that the core size of the QD was ~5-10 nm in diameter (Figure 1A, B, D, & E). EDX results show that QD-COOH and QD-NH_2_ have similar metal constituents: Cd, Se, Zn, and S (Figure 1C & F). There was an additional peak in the EDX of the QD-NH_2_ sample indicating the presence of phosphorous (P). Hydrodynamic diameter was reported by the manufacturer to be ~25 nm. Dynamic light scatterer (DLS) measurements of the particle prepared as the high dose for instillation gave a hydrodynamic particle/aggregate diameter range of 18.06-102.2 nm for QD-NH_2_ and 25.55-243 nm for QD-COOH with median diameters of ~29 nm and 50 nm, respectively. See Additional file 1 for percent distribution from DLS measurements. TEM images appeared to confirmed DLS size measurements, with particle/aggregates in PBS that consisted primarily of 1–5 QD for both samples, with a slightly greater percentage of larger aggregates in the QD-COOH sample.

**Figure 2:**
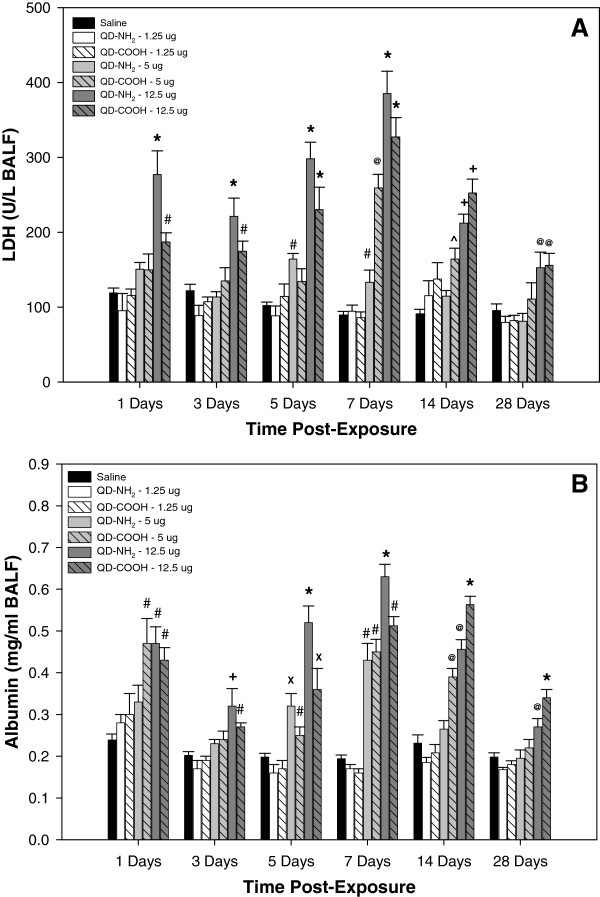
**Lung injury in vivo.** Indices of lung injury measured in the acellular BALF recovered from rats 1, 3, 5, 7, 14, and 28 days after intratracheal-instillation with 1.25, 5.0, or 12.5 μg of QD-NH_2_ or QD-COOH: (**A**) Lactate dehydrogenase (LDH), an indicator of general cytotoxicity, and (**B**) albumin, an indicator of damage to the air-blood barrier. * significantly different from all groups; ^+^ significantly different from saline, 1.25 μg, and 5.0 μg groups; ^#^ significantly different from saline and 1.25 μg groups; ^@^ significantly different from saline, 1.25 μg groups, and QD-NH_2_ at 5.0 μg; ^X^ significantly different from saline, 1.25 μg groups, and QD-COOH at 5.0 μg; ^ significantly different from saline (p<0.05).

Indices of lung injury were quantified in the recovered acellular bronchoalveolar lavage (BAL) fluid (BALF) at different time points after intratracheal instillation of different doses of the QD-NH_2_ and QD-COOH samples (Figure 2). LDH and albumin were significantly elevated after treatment with both QD samples at the 12.5 μg dose at each time point post-treatment up to 28 days, with slightly more injury resulting from the QD-NH_2_ treatment at days 1 through 5 post-treatment, and from the QD-COOH exposure at days 14. After treatment with the 5.0 μg dose, the QD samples caused an increase in both lung injury parameters, primarily at days 5, 7, and 14 after treatment. Treatment with the lowest dose (1.25 μg) did not result in significant lung injury compared to vehicle control.

**Figure 3:**
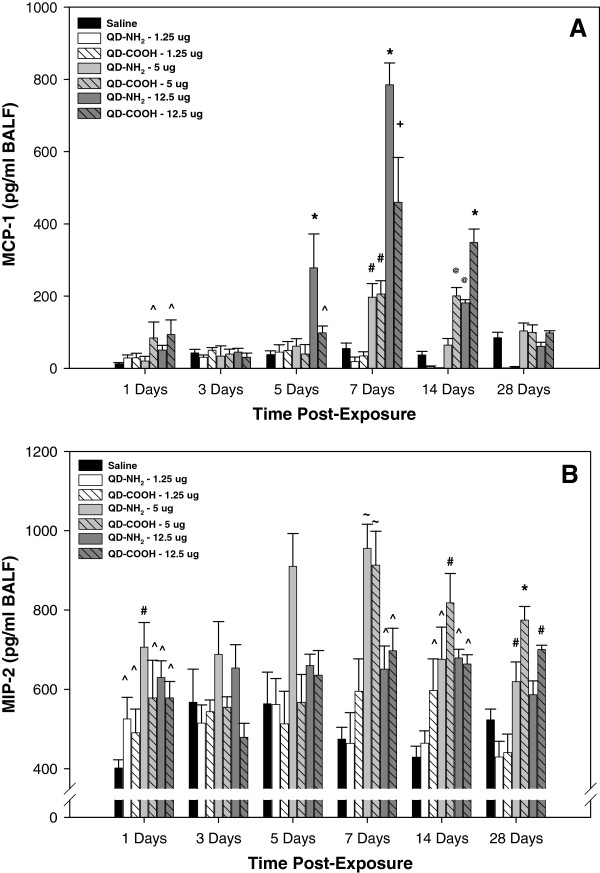
**In vivo pulmonary inflammatory indices.** Inflammatory chemokines, monocyte chemotactic protein (MCP)-1 (**A**) and macrophage inflammatory protein (MIP)-2 (**B**), were measured in the acellular BALF recovered from rats 1, 3, 5, 7, 14, and 28 days after intratracheal-instillation with 1.25, 5.0, or 12.5 μg of QD-NH_2_ or QD-COOH. * significantly different from all groups; ^+^ significantly different from saline, 1.25 μg, and 5.0 μg groups; ^#^ significantly different from saline and 1.25 μg groups; ^@^ significantly different from saline, 1.25 μg groups, and QD-NH_2_ at 5.0 μg; ^~^ significantly different from 12.5 μg dose, 1.25 μg dose, and saline; ^ significantly different from saline (p<0.05).

Inflammatory chemokines, MCP-1 and MIP-2, were measured in the recovered acellular BALF at different time points after intratracheal instillation of different doses of the QD-NH_2_ and QD-COOH samples (Figure 3). At 1 day, MCP-1 levels were significantly increased compared to the other groups after treatment with the 5.0 and 12.5 μg dose of the QD-COOH sample (Figure 3A). MCP-1 levels were significantly elevated after exposure to the 12.5 μg dose of both QD samples on days 5, 7, and 14 after treatment. The elevation was significantly greater in the QD-NH_2_ group when compared to the QD-COOH group at days 5 and 7, and greater in the QD-COOH group at day 14. A similar response was observed for 5.0 μg dose at 7 and 14 days, whereas no significant differences were observed between the groups at any of the time points after treatment with the 1.25 μg dose. MIP-2 concentrations were elevated in all groups when compared to vehicle control at day 1 and for the 5.0 and 12.5 μg doses at days 7, 14, and 28 after treatment (Figure 3B). At the time points after day 1, there was no significant difference in MIP-2 when comparing the 1.25 μg dose group and control.

**Figure 4:**
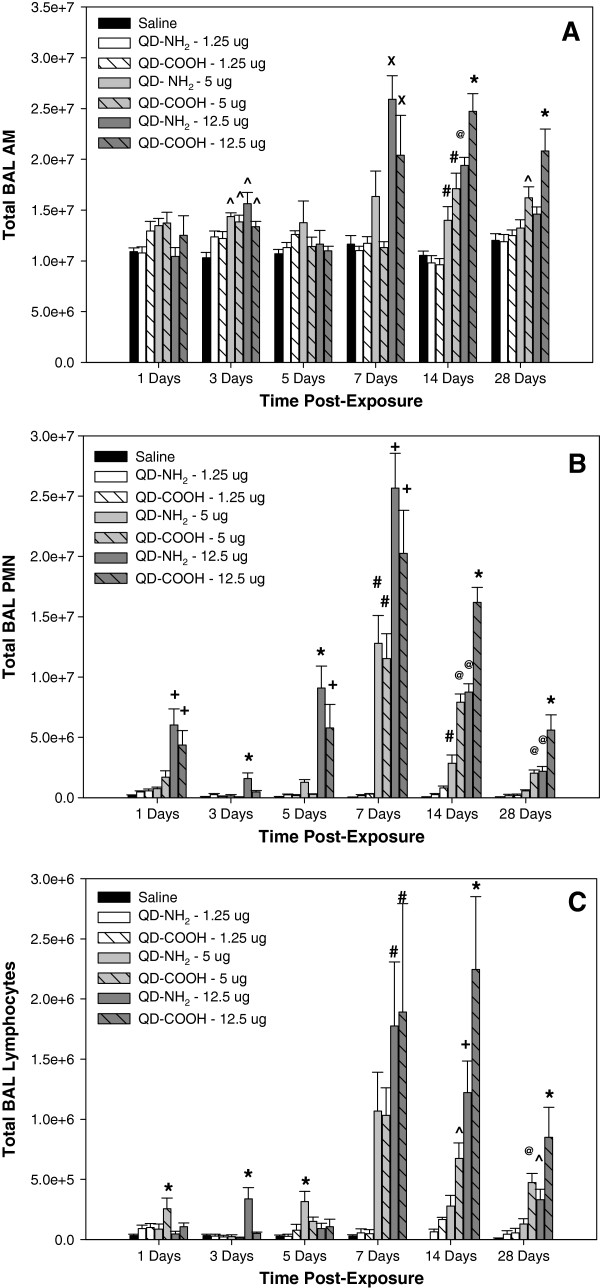
**Cellular differentials in bronchoalveolar lavage.** Total number of (**A**) alveolar macrophages (AM), (**B**) polymorphonuclear cells (PMN), and (**C**) lymphocytes recovered by from rats 1, 3, 5, 7, 14, and 28 days after intratracheal-instillation with 1.25, 5.0, or 12.5 μg of QD-NH_2_ or QD-COOH. * significantly different from all groups; ^+^ significantly different from saline, 1.25 μg, and 5.0 μg groups; ^#^ significantly different from saline and 1.25 μg groups; ^@^ significantly different from saline, 1.25 μg groups, and QD-NH_2_ at 5.0 μg; ^X^ significantly different from saline, 1.25 μg groups, and QD-COOH at 5.0 μg; ^ significantly different from saline (p<0.05).

Increases in specific inflammatory chemokines after treatment with the different QD samples correlated with inflammatory cells recovered by BAL at different time points after IT instillation (Figure 4). Elevations in lung AM were first observed at 3 days after treatment with 5.0 and 12.5 μg of both QD samples (Figure 4A). The number of AM recovered from the lungs peaked at 7 and 14 days and were significantly elevated at the highest dose of the QD samples. At 28 days after treatment, lung AM recruitment was still significantly elevated in the 5 and 12.5 μg dose QD-COOH groups. There was a significant increase in lung PMN in rats exposed to the 12.5 μg dose of both QD samples throughout the 28-day period with the peak response occurring at 7 and 14 days after treatment (Figure 4B). There was a greater number of PMN recovered from the lungs of rats treated with the highest dose of QD-NH_2_ compared to QD-COOH at 3 and 5 days, whereas inflammation was greater after treatment with the QD-COOH sample at 14 and 28 days. Both QD samples increased lung PMN numbers at 7 and 14 days after treatment with the 5.0 μg dose. Pulmonary treatment with the lowest dose (1.25 μg) had no effect on lung PMN number. Lymphocyte influx into the lung followed a similar pattern as PMN infiltration (Figure 4C).

**Figure 5:**
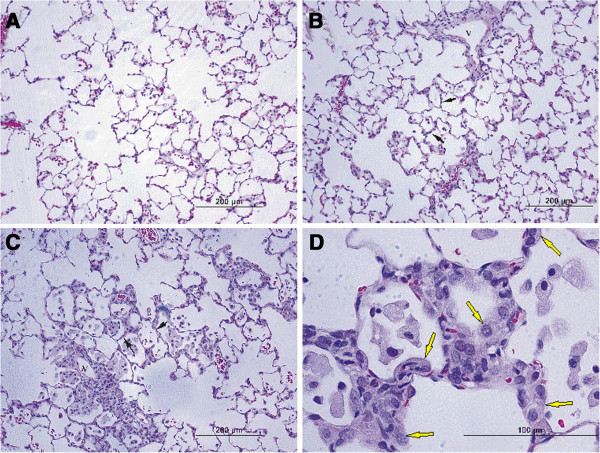
**Histopathological evaluation of pulmonary injury and inflammation.** Micrographs of H&E-stained tissue sections from rats 28 days after intratracheal instillation with saline (**A**), QD-NH_2_ (**B**), or QD-COOH (**C** &**D**). At 28 days after instillation of QD-NH_2_, infiltration of leukocytes around a blood vessel and in alveoli are present, with minimal thickening of alveoli septa (**B**, black arrows). At 28 days in the QD-COOH group, alveolar walls are thickened (**C**, black arrows) and alveoli contain foamy macrophages, and hyperplastic Type II epithelial cells can be found in alveoli (**D**, yellow arrows). V, vessel.

Histopathological evaluation was performed on lung tissue from rats 1, 7, 14, and 28 days after treatment with saline, QD-NH_2_, or QD-COOH. At 1 day post-instillation, there was minimal to mild inflammation (a score of 1–2) in QD-treated rats characterized by histiocytic and eosinophilic infiltration. A minimal level of epithelial cell necrosis and hypertrophy was present in the QD-NH_2_ group only (data not shown). This inflammation persisted up through 28 days post-exposure, peaking on days 7 and 14, and was slightly more pronounced in the QD-COOH group at days 7, 14, and 28. Alveolar septal thickening was present on days 14 and 28 (Figure 5B & C) in both groups, primarily due to infiltration of macrophages and granulocytes, and was particularly notable in the terminal bronchiolar region in the QD-COOH group. On day 28 in the QD-COOH group, foamy macrophages were found in alveoli and clustered around vessels (Figure 5D).

**Figure 6:**
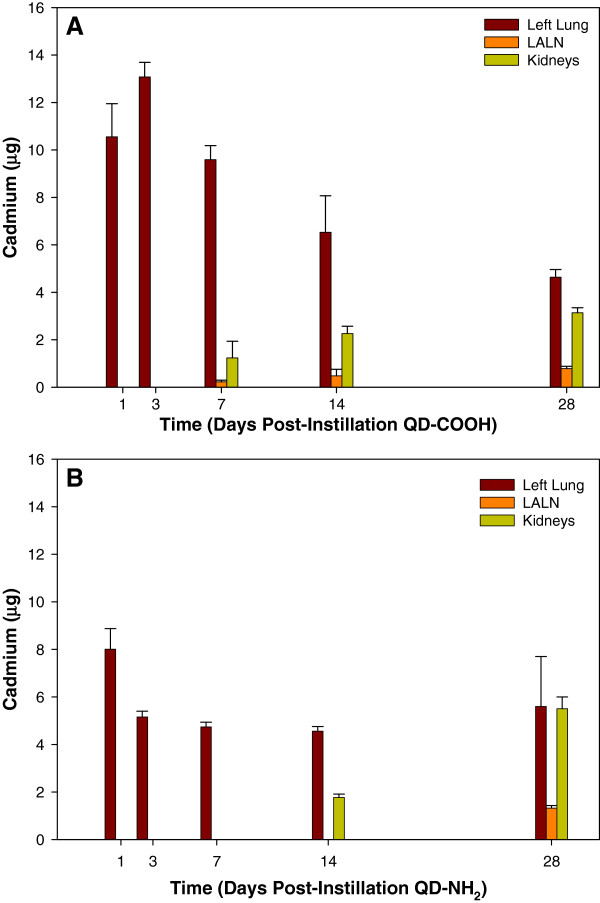
**Neutron activation analysis of QD biodistribution following pulmonary exposure.** Biodistribution of QD over time in rats intratracheally-instilled with 12.5 μg of QD-COOH (**A**) or QD-NH_2_ (B) using neutron activation to quantify the amount of cadmium (Cd) per organ. Organs measured were: left lung, lung-associated lymph nodes (LALN), blood, heart, liver, kidneys, brain, and spleen. No Cd was detected in the blood, heart, liver, spleen, or brain.

In the assessment of the biodistribution and clearance of Cd after QD treatment, the initial amount of Cd deposited in the left lungs of rats treated with QD-COOH (10.55 ± 1.4 μg) was approximately 20% more than that of those treated with QD-NH_2_ (8.01 ± 0.87μg) (Figure 6). Difference in distribution may likely be due to the route of exposure as intratracheal instillation can lead to an uneven distribution between left and right lung lobes; however, there may also be differences in the amount of Cd between the two forms of QD where there is slightly more Cd present in the COOH form as the left lung burden is approximately 13 μg on day 7. In addition, if it is assumed that the left lung represents half the Cd burden, there is approximately twice as much Cd in the samples as reported by the manufacturer indicating the actual high dose was closer to 20 μg rather than 12.5 μg, and possibly higher in the COOH sample.

Relative to the initial amount deposited, rats exposed to QD-COOH were able to clear more QD from the lungs over time when compared to rats exposed to QD-NH_2_; however, the majority of particles in both groups persisted out to day 14 in the lungs. No Cd was measured above the limit of detection in the blood, brain, heart, liver, or spleen in either treatment group (data not shown), either due to the lack of translocation or the relatively small amount of Cd that may have translocated relative to the overall organ weight due to the limit of detection in rats exposed to QD-COOH, CD appeared in the LALN and kidneys as early as day 7, and ~ 3-5% of Cd instilled could be detected in the LALN while ~10-20% was located in the kidney by day 14 (Figure 6A). By day 28, increased clearance from the lungs and increased deposition in LALN and kidneys was evident. The pattern of clearance for rats exposed to QD-NH_2_ was similar; however, the time frame for clearance to the kidney and LALN was slower. In rats treated with QD-NH_2_, ~20% of the instilled Cd was located in the kidneys by day 14, and no Cd was detected in the LALN until day 28 (Figure 6B).

**Figure 7:**
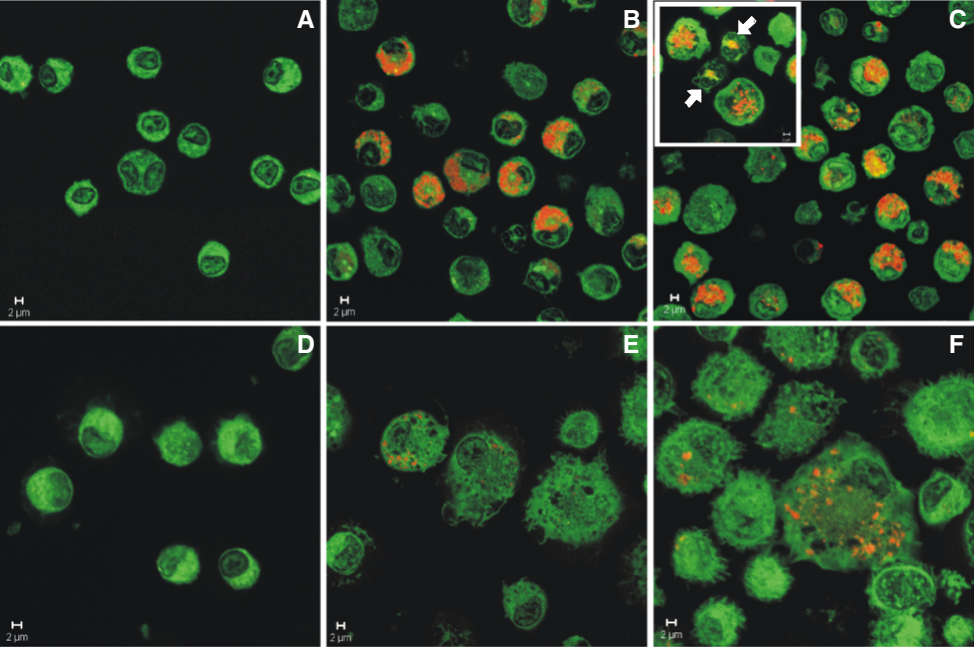
**Confocal evaluation of AM uptake of QD in vivo.** Confocal micrographs (63x-zoomed or 100x objective) of AM from the BAL of rats 1 (**A-C**) and 28 (**D-F**) days after intratracheal instillation with 12.5 μg QD-NH_2_ or QD-COOH (red/yellow), or PBS (control): (**A**) control – 1 day post-exposure, (**B**) QD-NH_2_ - 1 day post-exposure, (**C**) QD-COOH – 1 day post-exposure, (**D**) control – 28 days post-exposure, (**E**) QD-NH_2_ - 28 days post-exposure, and (**F**) QD-COOH – 28 days post-exposure. AM are green and QD are yellow/red. PMN uptake of QD is shown in the smaller inset micrograph in panel **C** (arrows).

Fluorescent confocal microscopy was used to image QD in lung cells recovered by BAL at different time points after treatment (Figure 7). Particles were concentrated inside AM at 1 day after lung treatment with 12.5 μg of either the QD-NH_2_ (Figure 7B) or QD-COOH (Figure 7C) sample and could be easily imaged up to 5 days post-exposure. This was also true for both QD samples at the 5.0 μg dose (data not shown). By day 28, very little QD in AM could be imaged using confocal microscopy (Figure 7E & F), possibly due to clearance and/or destabilization of the QD leading to decreased fluorescence. QD were not observed inside AM after instillation of the 1.25 μg dose (data not shown). PMN also were observed to have engulfed QD at early time points after treatment (Figure 7 inset, arrows).

**Figure 8:**
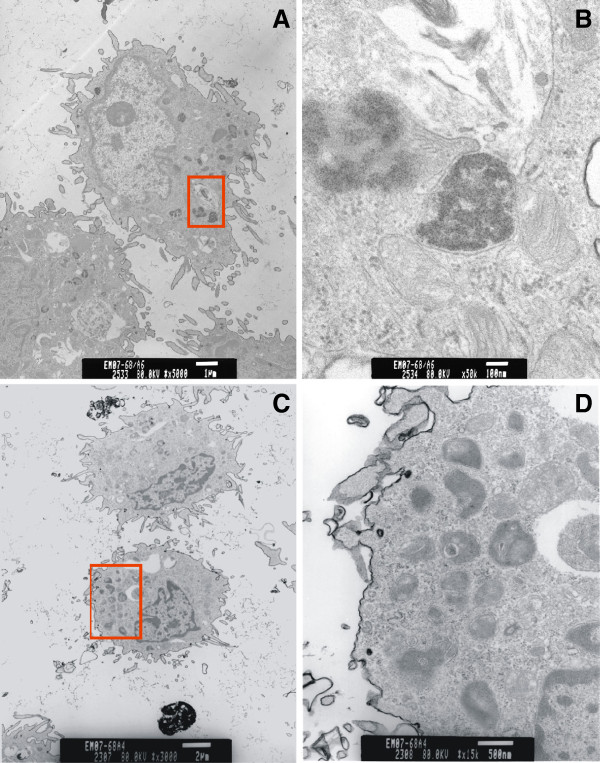
**AM uptake of QD visualized by transmission electron microscopy.** TEM micrographs of AM recovered by BAL at 1 day after intratracheal instillation with QD-COOH (**A **&**B**) or QD-NH_2_ (**C** &**D**) (12.5 μg/rat). High magnification TEMs of the area in the red box illustrate AM vacuoles containing QD-COOH (**B**) and QD-NH_2_ (**D**).

At 1 day after treatment, vacuoles containing QD-COOH or QD-NH_2_ were seen with high magnification TEM in AM recovered from BAL (Figure 8). EDX spectra confirmed that the vacuoles were positive for Cd, a core QD element. At the later time points (days 7, 14, and 28), it appeared that AM still contained QD; however, whether or not these vacuoles contain Cd, could not be confirmed with EDX (data not shown). It was interesting to note that AM from the rats treated with QD-COOH or QD-NH_2_ at the later time points post-treatment both contained “vacuoles” that had morphological structures that were not observed in AM from control rats (data not shown). The content of these crystal-like structures were not known.

**Figure 9:**
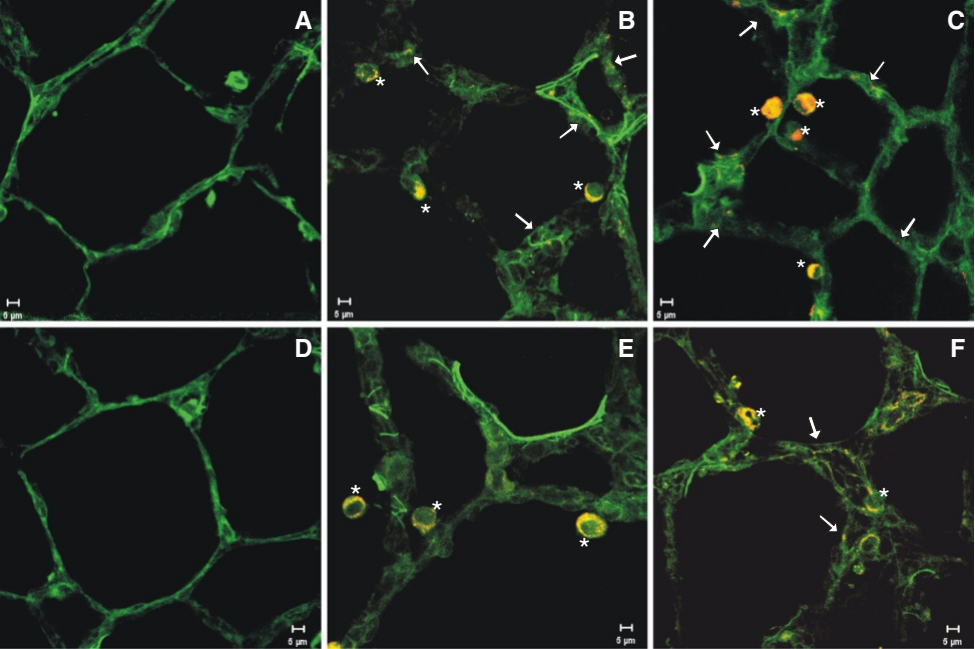
**Confocal evaluation of tissue distribution of QD.** Confocal micrographs of lungs (green) that have been cryo-preserved, sectioned, and imaged at 63x, 2 hours (**A**-**C**) and 1 day (**D**-**F**) after intratracheal instillation with 12.5 μg of QD-NH_2_, QD-COOH, or PBS (control): (**A**) control – 2 hours post-exposure, (**B**) QD-NH_2_ - 2 hours post-exposure, (**C**) QD-COOH – 2 hours post-exposure, (**D**) control – 1 day post-exposure, (**E**) QD-NH_2_ - 1 day post-exposure, and (**F**) QD-COOH – 1 day post-exposure. Lung tissue auto-fluoresces green and QD are yellow/red. * indicates QD-containing AM, white arrows indicate QD in tissue.

**Figure 10:**
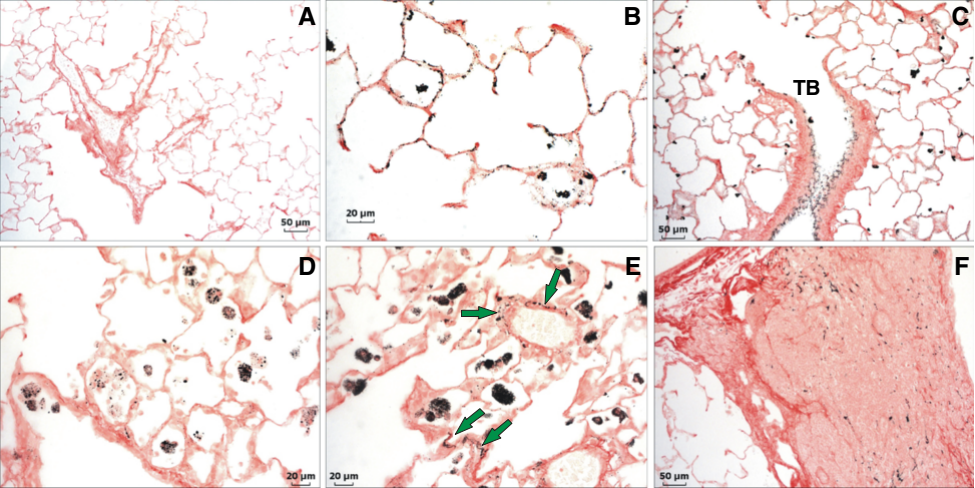
**Autometallographic evaluation of tissue distribution of QD 1 and 14 days post-exposure.** Silver enhancement staining of QD in lung tissue of rats 1 (**A**-**C**) and 14 (**E**-**F**) days after intratracheal instillation with 12.5 μg of QD-NH_2_ or QD-COOH, or PBS (control). (**A**) Lung from a control rat 1 day post-exposure showing no silver staining. (**B**) Silver enhancement of QD-COOH in the lung of a rat 1 day post-exposure. QD can be seen distributed along alveolar epithelium and in AM. (**C**) QD-NH_2_ enhanced with silver in rat lung 1 day after instillation. Distribution of QD-NH_2_ does not appear to differ drastically from QD-COOH 1 day post-exposure in terms of deposition in the alveolar region and in macrophages; however, there does appear to be slightly more deposition in the airways as shown in panel **C**. QD can also be seen throughout the epithelium of a terminal bronchiole (TB). By day 14, QD-NH_2_ (**D**) and QD-COOH (**E**) appear to be more concentrated in AM with a lesser amount in the tissue. QD-COOH can still be found in the interstitium and around vessels (green arrows). Both forms of QD were observed in bronchoalveolar lymphoid tissue (BALT). Silver enhancement of QD-COOH in BALT 14 days post-exposure is shown in panel **F**.

**Figure 11:**
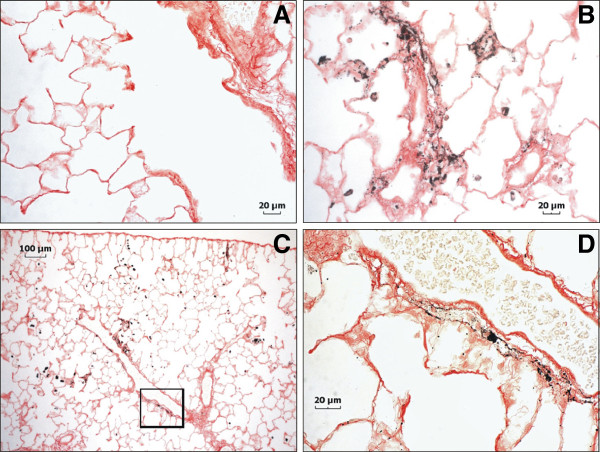
**Autometallographic evaluation of tissue distribution of QD 28 days post-exposure.** Silver enhancement staining of QD in lung tissue of rats 28 days after intratracheal instillation 12.5 μg of QD-COOH (**B**-**D**) or PBS (control) (**A**): a high magnification micrograph of QD-COOH showing AM clustered around vessels walls (**B**), a low magnification micrograph of a vessel with QD-COOH in and surrounding areas that may be perivascular lymphatic vessels (**C**), and several serial sections from the boxed area of Panel **C** imaged and stacked together at high magnification to illustrate accumulation of QD in and around a possible lymphatic vessel (**D**).

Lung tissue sections showing AM *in situ* that had phagocytized QDs were imaged by fluorescent confocal microscopy at 2 hr and 1 day after treatment (Figure 9). Numerous AM were observed to have engulfed QD early after treatment. Dispersed QD were observed throughout the alveolar region on the epithelium and in the interstitium (arrows) as late as day 5 after treatment. Because fluorescence associated with QD is highly dependent on the stability of the QD, an alternate imaging technique was employed to evaluate where particles persisted as instability may have affected evaluation by confocal microscopy. Silver enhancement staining of QD in lung tissue indicated that the QD in both treatment groups were found along airway epithelium, concentrated in AM, and deposited along the alveolar epithelium at 1 day after treatment (Figure 10A-C). Slightly more QD were deposited in the airways of rats treated with QD-NH_2_, whereas parenchymal deposition of QD in rats treated with QD-COOH was slightly greater. By day 14, QD were still found in AM, and, to a lesser degree, along the alveolar epithelium in rats from both groups (Figure 10D & E). QD were also found in bronchoalveolar lymphoid tissue (Figure 10F). Concentrations of QD in rats exposed to QD-COOH appeared to be located more in the interstitium and around vessels (arrows), in addition to being highly concentrated in AM. At 28 days after treatment, the QD-COOH sample was still readily detectable by silver enhancement (Figure 11), whereas the QD-NH_2_ silver-staining, although present, was not as intense (data not shown) despite persistence of Cd as indicated in Figure 6B. AM still contained a high concentration of QD- COOH, and they appeared to be concentrated around vessels (Figure 11B & C), possibly in the process of being cleared through the lymphatics from the lungs. A high magnification image illustrates the accumulation of the QD in and around a lymphatic vessel (Figure 11D).

## Discussion

QD are being developed and used for a variety of applications (e.g., drug delivery, diagnostic procedures) that may lead to human exposure in medical settings. In addition, the number of workers producing or using nanomaterials, such as QD, is growing substantially. Unfortunately, little information exists about the potential health hazards associated with medical, environmental, or occupational lung exposures to QD. One important occupational health issue that is being addressed by the National Institute for Occupational Safety and Health (NIOSH) Nanotechnology Research Center is determining the toxic potential and fate of nanomaterials in the body after exposure [13]. Therefore, there is interest in assessing the potential adverse health outcomes that may occur following pulmonary QD exposure, and the mechanisms underlying these responses. NIOSH is actively performing field studies to evaluate the potential exposure of workers in the QD industry (personal communication with Kenneth Martinez, 2012).

*In vivo* animal studies assessing the pulmonary responses after exposure to QD are limited. The QD samples examined in the current study were composed of a CdSe core. Cd is a widespread environmental and occupational pollutant and has been classified as a type I carcinogen by the International Agency for Cancer Research (IARC [14]). Inhalation of Cd-containing pollutants can result in metal fume fever, chemical pneumonitis, pulmonary edema, and pleural effusion [15]. In addition to composition and the presence of potentially toxic metals, surface coating, charge, and size may influence QD toxicity. *In vivo* lung toxicology studies have shown that nanoparticles of identical composition and mass may be more toxic (Brown et al. [16,17]) when compared to their larger counterparts. *In vitro* studies indicate that Cd-containing QD with a smaller diameter had greater toxicity and distributed to the nucleus of the cell compared to a larger QD of the same composition that were less cytotoxic and remained in the cell cytoplasm [18]. Functionality and surface charge of QD are also of important in regard to conjugation to other particles or molecules for targeted delivery of substances in the body or tracking of substances. The QD in the current study were functionalized with different surface moieties by the manufacturer to examine the effect of surface charge on toxicity and were determined to be in the ultrafine size range with core size diameters of 5–10 nm.

A significant increase in lung injury was observed at different time points after a single intratracheal instillation of the QD-COOH and QD-NH_2_ samples in the current study. Lung injury parameters, LDH and albumin, were significantly increased immediately after treatment with the 12.5 μg dose, peaked at day 7 post-exposure, and remained elevated throughout the 28 day period, even though the response at 28 days was subsiding. Evidence for lung injury was not apparent until 5 days after treatment with the 5 μg dose of QD-NH_2_ sample where it was found to be more potent than the QD-COOH treatment. At 7 days post-exposure, the 5 μg dose of the QD-COOH sample began to exhibit toxicity and was more potent than the QD-NH_2_ sample. The difference in temporal response between the two doses may be related to the lungs responding earlier to a substantially greater number of particles at the higher 12.5 μg dose (e.g., dose effect), whereas the later injury response after treatment with the 5.0 μg dose may better reflect a potential mechanism of toxicity, the *in situ* dissolution of the QD by AM, leading to the direct exposure of the lung cells to free Cd (e.g., chemical effect). Moreover, the QD-NH_2_ sample induced significantly more lung injury at earlier time points after treatment compared to the QD-COOH, which may be due to a difference in particle distribution related to charge or a slightly faster disruption of the QD-NH_2_ structure, allowing for cellular exposure to free Cd sooner, or a combination of the two. Multiple studies have indicated that coating affects destabilization of QD and that uncoated Cd-containing QD released free Cd from the QD core intracellularly and were cytotoxic *in vitro*[19,20]. In addition, Derfus et al. [21] demonstrated that the encapsulation of Cd/Se QD by a ZnS shell greatly reduced both the amount of free Cd released and the production of free radicals by QD.

In terms of lung inflammation as determined by the number of PMN recovered from the lungs, a significant inflammatory response was observed at 1 day after treatment with the 12.5 μg dose, likely due to a dose/bolus effect because this initial response subsided by 3 days after treatment, before increasing again and peaking at day 7. In addition, the lower dose QD treatment groups had no effect on inflammation at 1, 3, and 5 days after treatment. As was seen with the lung injury response, the number of recovered lung PMN was significantly greater at earlier time points (days 3 and 5) after treatment 12.5 μg dose of QD-NH_2_ sample compared to the QD-COOH sample, whereas PMN influx was greater in the QD-COOH-treated rats at days 14 and 28 when compared to those treated with QD-NH_2_, again implicating a likely difference in the dissolution rate between the two QD samples and the release of free Cd. Despite these differences, the pattern of inflammation is similar between QD groups, with peak responses between 7–14 days after treatment for the 5 and 12.5 μg dose groups for both QD samples. In addition to PMN influx, AM infiltration peaked at these two time points as well. In agreement with the inflammatory response seen in the current study, Ma-Hock et al. [10] also observed a transient local PMN inflammation in the lungs of rats that was associated with increases in inflammatory chemokines, MCP-1 and MIP-2, after a short-term inhalation of Cd-containing QD (cadmium sulfide core with a cadmium hydroxyl shell – CdS/Cd(OH)_2_)). This delay in inflammatory cell influx into the lungs after treatment is additional evidence that free Cd released from the dissolution of the QD structure is likely a causative factor responsible for the pulmonary responses observed. The lung inflammation pattern was similar to that of lung injury and appeared to be transient with the response subsiding (even at the highest dose) by 28 days after treatment with both QD samples.

Regarding lung deposition and clearance, QD-COOH and QD-NH_2_ were both rapidly phagocytized *in vivo* by AM in a dose-dependent manner as early as 2 hr after intratracheal instillation. Cd burden in LALN beginning 1 week after exposure suggests that at least a portion of the QD in the alveolar region are cleared via scavenging phagocytes which then migrate out of the lung through the lymphatics. Ma-Hock et al. [10] also observed AM clearance to the LALN as well. Translocation from the lung and clearance via the blood is also a possible route of clearance for particles in the lung. Confocal microscopy revealed some QD to have deposited on the epithelium outside AM and move to the interstitium, giving this portion of QD the potential of reaching the systemic circulation and translocating to other organs. However, measureable levels of Cd as determined by neutron activation did not appear in a systemic organ until 7 and 14 days after treatment with the QD-COOH and QD-NH_2_ samples, respectively. Using an *in vitro* model, Geys et al. [22] examined the effect of surface charge on the translocation of non-functionalized QD, QD-COOH, and QD-NH_2_ through a tight monolayer of primary rat alveolar epithelial cells. They observed that QD did not translocate through the tight junctions of alveolar epithelial cells, regardless of surface charge, without disruption of the cell-to-cell barrier induced by oxidative stress. In the current study, Cd depositing in extrapulmonary organ systems corresponds to the time points post-exposure where the greatest lung injury is observed, rendering it likely that the translocation of QD is occurring, and that this may be a passive process via “leaky” cell junctions, rather than an active process.

Neutron activation showed that at day 1 there was more Cd measured in the lungs of rats treated with QD-COOH when compared to those treated with QD-NH_2._ Uneven distribution due to route of administration (intratracheal instillation resulting in more QD going to one bronchus or the other), differences in deposition pattern (greater deposition in upper airways versus parenchyma), or differences in destabilization and translocation of the particles in the first 24 hours may contribute to this discrepancy. Differences in the rate of clearance were evident with greater percentage of QD cleared from the lungs by day 28 in the QD-COOH group compared to the QD-NH_2_ group, relative to early deposition amounts. However, the pattern of clearance via the lymphatics, and translocation to the kidneys via the blood stream, were similar between the two QD, and neutron activation analysis showed that Cd persisted in the lungs at least 28 days after exposure for both QD. This data is in agreement with other studies. Using inductively coupled plasma optical emission spectroscopy (ICP-OES) as opposed to neutron activation, Ma-Hock et al. [10] also observed that Cd levels in the lungs did not decline during a 3-week recovery period after inhalation exposure to water soluble CdS/Cd(OH)_2_.

Silver enhancement staining of lung tissue recovered from the QD-treated animals in the current study provided additional evidence that Cd persisted in the lungs at 28 days after treatment. The silver staining, used to image deposited metal particles, taken into consideration with the diminished fluorescent signal observed 1 week after exposure to QD using confocal microscopy, is another indication that free Cd had dissociated from the QD beginning 5–7 days after treatment. Although both forms of QD persisted in the lung up to 28 days, the rate of clearance appears to differ between the positively and negatively charged particles, with a slower clearance rate observed for QD-NH_2_ particles. This difference in the clearance patterns may be due to different dissolution rates of Cd from the QD after AM phagocytosis and enzymatic digestion because of the different surface charges and properties of the two QD samples as discussed above. In addition, a greater influx of phagocytes was observed at the later time points post-exposure to QD-COOH when compared to QD-NH_2_, resulting in a greater capacity for clearance. Also, it should be noted that initial Cd deposition in the lungs was greater with the QD-COOH sample compared to the QD-NH_2_ sample and this may contribute to a greater initial clearance of QD-COOH. As mentioned above, for both QD, a portion of the clearance appeared to occur via mechanical clearance by AM, as well as by extrapulmonary translocation.

The kidney, a primary target organ of Cd toxicity, was the only extrapulmonary organ in which Cd was measured in the current study. Relatively small concentrations of Cd appeared in the kidneys at 7 days after QD-COOH treatment and at 14 days after QD-NH_2_ treatment with levels increasing at 28 days for both groups. Ma-Hock et al. [10] also observed small amounts of Cd in kidney, which increased very slightly over the post-exposure recovery period. Importantly, histological analysis of kidney in their study did not indicate any morphological abnormalities after short-term QD inhalation. The Cd levels measured in the kidney of the current study were substantially lower than the levels of 104–252 μg/g wet kidney that were observed to cause renal dysfunction in rats after oral Cd treatment [23]. The presence of Cd in the kidney is in line with the findings of Chen et al. [24] and Lin et al. [25] who observed the accumulation of QD in the kidney of rats and mice, respectively, after intravenous injection. However, Ma-Hock et al. [10] measured Cd in the liver after inhalation, and both Chen et al. [24] and Lin et al. [25] observed QD in the liver after intravenous exposure. Measureable levels of Cd were not detected in the liver after intratracheal treatment of the different QD samples in the current study, likely due to dose and route of administration combined. In agreement with the Ma-Hock et al. [10] study, lung treatment did not lead to the accumulation of Cd in the brain or spleen in the current study.

Based on the findings of the current study and others, several conclusions can be made concerning the toxicity and fate of QD after pulmonary treatment. Lung exposure to high enough concentrations of CdSe QD can cause transient lung inflammation and injury that appears to be related to the dissolution of the QD structure and removal of the ZnS shell coating, allowing for the release of free Cd. Different surface charges due to the addition of different functional groups attached to the surface of the QD may influence both the toxic pulmonary response and the persistence and clearance of the Cd from the lungs. QD-NH_2_ appeared to induce more injury and inflammation at earlier time points post-exposure where epithelial cell necrosis and type II cell hyperplasia were minimal but present; whereas QD-COOH cause greater injury and cellular influx at the later time points post-exposure. Although differences exist between the two particles, the pattern of toxicity was primarily the same for both QD with toxicity peaking at days 7 and 14 and beginning to subside over time. This is in agreement with Clift et al. [11], who found that QD with organic coatings were more toxic than QD-COOH and QD-NH_2_(PEG) with only slightly more toxicity present with the carboxyl groups versus the amine groups. In addition, toxicity appears to be dependent on the availability of free Cd for both QD, as well, although dissolution rates may vary. Overall, despite a relatively high pulmonary load, the systemic availability of the QD appears to be low. No free Cd was measured in blood, brain, liver, and heart in the current study. Measureable, but relatively small, amounts of free Cd did accumulate in the kidney, the main storage organ of Cd in the body.

Overall, both forms of QD induced lung damaged which was more severe one week after exposure, with QD-NH_2_ having induced more toxicity at the early time points post-exposure and QD-COOH having been more toxic at the later time points. However, the peak damage for both QD is around day 7 and sustained until day 14. This damage coincided with the lack of fluorescence in lung tissue under confocal microscopy, which indicates destabilization of the QD, and with evidence that Cd still persisted in the lungs (silver staining of tissues) at the times of peak lung damage. The data suggests that Cd became bioavailable over time due to destabilization of the QD in the lung, and induced injury as it became available and while it persisted there. Althought the rate of clearance of the QD-NH_2_ and QD-COOH differed, indicating a potentially different destabilization rate, the pattern of clearance over time was similar, primarily via the lymphatics and translocation systemically to the kidneys.

## Methods

### Quantum dots and characterization

Water-soluble quantum dots (Evident Technologies, Inc., Troy, NY) (QD), with a hydrodynamic diameter in the range of 30–50 nm, were composed of a CdSe core with a ZnS shell and had an emission peak of 620nm. The surface of the QD particles was functionalized with either carboxylic terminal groups (QD-COOH; stock = 2.1 nmol/ml water equivalent to 250 μg/ml) or amine terminal groups (QD-NH_2_; stock = 3.4 nmol/ml water equivalent to 250 μg/ml) by the manufacturer and were supplied in water (no toluene present). Transmission electron microscopy (TEM) was performed to detect individual and agglomerated QD in suspension. QD were diluted in deionized water to a concentration of 2.5 μg/ml and evaluated using a JEOL-1220 transmission electron microscope (JEOL, Inc., Tokyo, Japan). In addition QD suspended in PBS at a final instillate concentration were also imaged. Field emission scanning electron microscopy (FESEM) was used to obtain an energy dispersion X-ray (EDX) to illustrate elemental composition. For FESEM, QD were suspended in deionized water at a concentration of 2.5 μg/ml, dried onto carbon planchets, and imaged using a Hitachi Model S-4800 microscope (Hitachi High Technologies America, Inc., Gaithersburg, MD) operated between 5 and 20 kV. In addition, QD suspended in phosphate-buffered saline (PBS) at the instillate concentration for groups exposed to the high dose of particles were also analyzed using a dynamic light scatterer (DLS; Microtrac Inc., Largo, FL) to assess aggregation in the delivery vehicle. TEM of the instillate was also performed to further analyze aggregation of particles.

### Animals

Male Sprague–Dawley [Hla:(SD)CVF] rats (Hilltop Laboratories, Scottdale, PA) weighing 250–300 g, approximately 10 weeks old, were used for all experiments in accordance with a protocol approved by the institute’s Animal Care and Use Committee (ACUC). Animals were given a Teklad 2918 diet and tap water *ad libitum*, housed in a clean air and viral- and antigen-free room with restricted access in an Association for Assessment and Accreditation of Laboratory Animal Care International (AAALAC)-approved animal facility. All animals were allowed to acclimate for one week before use. The rats were monitored and found to be free of endogenous viral pathogens, parasites, mycoplasms, Helicobacter, and CAR Bacillus.

### *In vivo* exposure and study design

On day 0, male Sprague–Dawley rats were lightly anesthetized by an intraperitoneal (i.p.) injection of 0.6 ml of a 1% solution of sodium methohexital (Brevital, Eli Lilly, Indianapolis, IN), and intratracheal instillation (IT) was performed with a volume of 300 μl of QD-NH_2_ or QD-COOH diluted in PBS at doses of 12.5 μg, 5.0 μg, or 1.25 μg /rat. Vehicle control rats received an equivalent volume (300 μl) of sterile PBS by IT. Treated animals then were sacrificed with an overdose of sodium pentobarbital (>100 mg/kg bwt; Sleepaway, Fort Dodge Animal Health, Wyeth, Madison, NJ) followed by exanguination at 1, 3, 5, 7, 14, or 28 days after QD exposure. An additional 2 hour time point was conducted with the 12.5 μg dose to evaluate lung tissue for QD deposition and biodistribution. Bronchoalveolar lavage (BAL) was performed on right lungs with one lobe ligated for later microscopic preparation and analysis. To assess lung damage, lactate dehydrogenase (LDH) and albumin levels were measured in the first fraction of acellular BAL fluid (BALF). Recovered BAL cells were differentiated to assess lung inflammation, as well as seeded onto glass coverslips and fixed for laser scanning confocal microscopy (LSCM) analysis. Additional aliquots of BAL cells were suspended in Karnovsky’s fixative and imaged using TEM. The ligated right lobe of rats treated with 12.5 μg of QD was either: (1) inflated with a Tissue-Tek O.C.T. Compound (Sakura Finetek Inc., Torrance, CA) + 20% sucrose solution, cryo-preserved, and sectioned for LSCM or (2) pressure inflated with 10% formalin, embedded, sectioned, and stained for histological evaluation or autometallographic silver enhancement. To assess biodistribution and clearance of QD, left lungs, lung-associated lymph nodes (LALN), blood, brains, hearts, kidneys, livers, and spleens were analyzed using neutron activation.

### BAL cell and fluid collection

BAL was performed by washing the lungs of treated rats with aliquots of PBS in order to obtain pulmonary cells for morphological and functional analysis. The acellular BALF was retained for analysis of indicators of tissue damage and cellular activity. Rats were euthanized with an i.p. overdose of sodium pentobarbital (>100 mg/kg body weight; Sleepaway, Fort Dodge Animal Health, Wyeth, Madison, NJ), the trachea was cannulated, the chest cavity was opened, the left lung was clamped off, one right lobe was ligated, and BAL was performed on the right lung via the tracheal cannula at different time points after IT. The acellular fraction of the first BAL was obtained by filling the right lung with 1 ml/100 g body weight of PBS, massaging for 30 seconds, withdrawing, and repeating the process one more time. This concentrated aliquot was withdrawn, retained, kept separately, and was designated as the first fraction of BALF. The following lavage aliquots were 6 ml in volume, instilled once with light massaging, withdrawn, and combined until a 30 ml volume was obtained. For each animal, both fractions of BAL were centrifuged, the cell pellets were combined and resuspended in 1ml of PBS, and the acellular fluid from the first fraction was retained for further analysis.

### Evaluation of lung injury and inflammation

#### LDH and albumin

The presence of albumin and LDH in the acellular BALF of all treatment groups was measured to evaluate the loss of integrity of the alveolar-capillary barrier and general cytotoxicity, respectively. Measurements of both albumin and LDH in the acellular fluid were obtained using a Cobas Mira analyzer (Roche Diagnostic Systems, Montclair, IN). Albumin was determined by spectrophotometric measurement of the reaction product of albumin with bromcresol green (628 nm) according to a method by Sigma Diagnostics (St. Louis, MO). LDH activity was quantified by detection of the oxidation of lactate coupled to the reduction of NAD+ at a spectrophotometric setting of 340 nm.

#### Inflammatory chemokines

Monocyte chemotactic protein (MCP)-1 and macrophage inflammatory protein (MIP)-2 were measured in the acellular BALF of rats pre-treated with QD or PBS using commercially available enzyme-linked immunosorbent assay (ELISA) kits (BioSource International Inc., Camarillo, CA).

#### Cell differentials

Total BAL cells collected from rats treated with QD or PBS were counted using a Coulter Multisizer II (Coulter Electronics, Hialeah, FL). Cell differentials were performed to determine the total number of AM, polymorphonuclear leukocytes (PMN), and lymphocytes. Briefly, 1 × 10^5^ cells from each rat were spun down onto slides with a Cytospin 3 centrifuge (Shandon Life Sciences International, Cheshire, England) and labeled with Leukostat stain (Fisher Scientific, Pittsburgh, PA) to differentiate cell types. Two hundred cells per slide were counted, and the percentage of alveolar macrophages (AM), polymorphonuclear cells (neutrophils, PMN), and lymphocytes were multiplied by the total number of cells to calculate the total number of each cell type.

#### Histopathology

The formalin fixed right lobe from animals in each group was embedded in paraffin, sectioned onto slides, and stained with hematoxylin and eosin (H&E). H&E-stained slides were qualitatively analyzed for indications of inflammation and injury by a certified veterinary pathologist at Charles River Laboratories (Wilmington, MA) who was blinded to the treatment groups. Indices of inflammation and injury were scored on scale of 0–5, where 0 = no observed effect, 1 = minimal response, 2 = mild response, 4 = moderate response, and 5 = severe response.

### QD lung deposition, clearance, and biodistribution

#### Neutron activation

Left lung, LALN, blood, brain, heart, kidney, liver, and spleen tissue were collected from a set of animals from each treatment group (n = 3–4). Tissues were freeze-dried by lyophilization, weighed, and analyzed for Cd content by North Carolina State University Nuclear Reactor Program, Department of Nuclear Engineering (Raleigh, NC) using neutron activation. Briefly, tissue samples and standards were irradiated in a PULSTAR reactor rotating exposure port for 12 MW-hr. Samples decayed for approximately 1 wk and were counted for 10 min each on a gamma spectroscopy system analyzing for Cd.

### Confocal microscopy

Cryo-preserved lung tissue was sectioned, and lung tissue sections and BAL cells were imaged with a LSCM (Carl Zeiss, Inc., Thornwood, NY). Tissue sections were scanned at 63x with a 543nm laser and an LP585 filter to detect the QD (red), and tissue autofluorescence was detected with a 488nm laser (green). Cells were counter-stained with CellTracker green (Invitrogen Corp., Carlsbad, CA) and imaged with a 63x or 100x objective at similar laser settings.

### Electron microscopy

BAL cells were preserved with Karnovsky’s fixative, post-fixed in osmium tetroxide, and embedded in epoxy resin. QD were imaged in AM using a JEOL 1220 transmission electron microscope (JEOL, Inc., Tokyo, Japan) at 80 KV.

### Tissue autometallographic silver enhancement

For light microscope visualization of QD, paraffin-embedded lung tissue sections were deparaffinized and dehydrated. The tissue sections were then washed with multiple changes of deionized water. Sections were developed with a silver enhancement kit according to the manufacturer’s instructions (Polysciences, Inc., Warrington, PA). The reaction was terminated after 30 min of development by washing in deionized water, and then the lung tissue sections were lightly counter-stained with Sirius Red [26].

### Statistical analysis

Results are expressed as means ± standard error of measurement (SE). Statistical analyses were carried out with the SigmaStat 3.1 statistical program (Chicago, IL). The significance of the interaction among different treatment groups for the different parameters at each time point was assessed using analysis of variance (ANOVA). The significance of difference between individual groups was analyzed using the Holm-Sidak method with the criterion of significance set at *p*<0.05.

## Competing interests

There are no financial or non-financial competing interests.

## Authors’ contributions

JRR contributed to the experimental design of the study, performed animal procedures analyzed data from animal studies, and drafted manuscript. JMA assisted in experimental design, exposed animals, and aided in drafting the manuscript. DWP and VC contributed to the experimental design and data analysis. RSC conducted animal and in vitro procedures and analyzed data from bronchoalveolar lavage. SHY conducted in vitro studies and performed flow cytometry. DSB designed and conducted electron microscopy studies. JFC and RRM designed, performed, imaged and analyzed autometallography studies, and aided in tissue distribution analysis. All authors have approved the final version of the manuscript.

## Disclaimer

The findings and conclusions in this report are those of the authors and do not necessarily represent the views of the National Institute for Occupational Safety and Health.

## Supplementary Material

Additional file 1 **Percent size distribution of QD particles/aggregates measured by dynamic light scattering (DLS).** DLS measurements of the hydrodynamic diameter size distribution of the high dose preparation of QD-COOH and QD-NH_2_ particles and aggregates in PBS. Diameters ranged from 18.06-102.2 nm for QD-NH_2_ and 25.55-243 nm for QD-COOH, with median diameters of approximately 29 nm and 50 nm, respectively. Click here for file
