# Supplementary material for: Lung toxicity and biodistribution of Cd/Se-ZnS quantum dots with different surface functional groups after pulmonary exposure in rats
Source: Part Fibre Toxicol. 2013 Mar 4;10:5. doi: 10.1186/1743-8977-10-5 (PMC3599433; doi:10.1186/1743-8977-10-5)
Supplement: Additional file 1 — Percent size distribution of QD particles/aggregates measured by dynamic light scattering (DLS). DLS measurements of the hydrodynamic diameter size distribution of the high dose preparation of QD-COOH and QD-NH2 particles and aggregates in PBS. Diameters ranged from 18.06-102.2 nm for QD-NH2 and 25.55-243 nm for QD-COOH, with median diameters of approximately 29 nm and 50 nm, respectively. [file 1743-8977-10-5-S1.pdf]

Additional File 1.

Percent Size Distribution of QD Particles/Aggregates Measured by Dynamic Light Scattering (DLS)

$QD-NH_2$			QD-COOH	
**Diameter (nm)**	**% of Sample**		**Diameter (nm)**	**% of Sample**
102.2	0.68		243	0.89
85.9	3.18		204.4	3.38
72.3	6.8		171.9	7.13
60.8	8.52		144.5	8.93
51.1	8.46		121.5	7.84
43	8.55		102.2	5.91
36.1	9.7		85.9	4.57
30.4	11.8		72.3	4.11
25.55	13.96		60.8	4.45
21.48	15.05		51.1	5.58
18.06	13.3		43	7.55
			36.1	10.35
			30.4	13.67
			25.55	15.64
**Median Hydrodynamic Diameter= ~29 nm**			**Median Hydrodynamic Diameter= ~50 nm**	
